# Induction of autophagy and autophagy‐dependent apoptosis in diffuse large B‐cell lymphoma by a new antimalarial artemisinin derivative, SM1044

**DOI:** 10.1002/cam4.1276

**Published:** 2017-12-26

**Authors:** Chunyan Cheng, Tao Wang, Zhiqun Song, Lijun Peng, Mengqing Gao, Olivier Hermine, Sophie Rousseaux, Saadi Khochbin, Jian‐Qing Mi, Jin Wang

**Affiliations:** ^1^ State Key Laboratory for Medical Genomics Department of Hematology Shanghai Institute of Hematology Collaborative Innovation Center of Systems Biomedicine Pôle Sino‐Français des Sciences du Vivant et Genomique Rui Jin Hospital Shanghai Jiao Tong University School of Medicine Shanghai China; ^2^ Department of Blood Transfusion the First Affiliated Hospital of Nanjing Medical University Nanjing Jiangsu China; ^3^ Service d'Hématologie Adultes Hôpital Necker‐Enfants Malades Assistance Publique‐Hôpitaux de Paris Université Paris Descartes Paris France; ^4^ CNRS UMR 5309/INSERM U1209/Université Grenoble‐Alpes/Institute for Advanced Biosciences La Tronche France

**Keywords:** Apoptosis, artemisinin derivative, autophagy, DLBCL, Survivin

## Abstract

Diffuse large B‐cell lymphoma (DLBCL) is the most common form of non‐Hodgkin's lymphoma. R‐CHOP is currently the standard therapy for DLBCL, but the prognosis of refractory or recurrent patients remains poor. In this study, we synthesized a new water‐soluble antimalarial drug artemisinin derivative, SM1044. The treatment of DLBCL cell lines with SM1044 induces autophagy‐dependent apoptosis, which is directed by an accelerated degradation of the antiapoptosis protein Survivin, via its acetylation‐dependent interaction with the autophagy‐related protein LC3‐II. Additionally, SM1044 also stimulates the de novo synthesis of ceramide, which in turn activates the CaMKK2–AMPK–ULK1 axis, leading to the initiation of autophagy. Our findings not only elucidate the mechanism of autophagy‐dependent apoptosis in DLBCL cells, but also suggest that SM1044 is a promising therapeutic molecule for the treatment of DLBCL, along with R‐CHOP regimen.

## Introduction

Diffuse large B‐cell lymphoma (DLBCL) is a common aggressive malignancy of mature B cells, accounting for 30–40% of adult non‐Hodgkin's lymphoma (NHL). R‐CHOP (rituximab and CHOP regimen) is currently the most approved standard of care for DLBCL, which significantly improved the therapeutic effects and prognosis of patients. Unfortunately, approximately 10–15% of cases are refractory to the treatment, and 20–30% of responders relapse 1. Even worse, roughly 50% of the relapsed cases are not eligible for high‐dose chemotherapy with a subsequent autologous stem cell transplantation (ASCT) 2. Thus, the prognosis of patients with relapsed or refractory DLBCL is still poor. International prognostic index (IPI) has long been established as the main prognostic indicator for aggressive NHL, including DLBCL. However, IPI only includes clinical parameters, and not biological indicators. Accumulating evidence suggests that molecular markers, such as high expression of Survivin, could be associated with poor prognosis DLBCL cases 3, 4.

Survivin is the smallest member of the inhibitors of apoptosis protein (IAPs) family, highly conserved during evolution, and is involved in the regulation of both cell division and apoptosis. Survivin has increasingly been recognized as a tumor gene because it is rarely expressed in normal differentiated tissues, while highly expressed in various cancers and mostly correlated with inferior outcomes 5, 6. Positive Survivin has also been closely related to tumor progression and chemoresistance 5, 7. In DLBCL, Survivin is overexpressed in up to 60% of the cases, and is associated with advanced clinical stage, reduced remission rate, shortened event‐free survival, as well as increased chemoresistance and relapse 8, 9. In addition, Survivin‐positive DLBCL patients seem to benefit less from R‐CHOP regimen compared to Survivin‐negative cases 4.

Interestingly, the knockdown of Survivin leads to significant apoptosis and growth inhibition of DLBCL cell lines, while its overexpression protects the cells from death 10, 11. In recent years, great efforts have been employed to test the feasibility and effectiveness of Survivin inhibition in treating tumors 12, 13. Currently, the selective Survivin inhibitor YM155 has been well validated in vitro and in vivo, and several clinical trials are ongoing to test its efficacy in patients 14, 15, 16. Thus, the downregulation or suppression of Survivin could be an attractive strategy for the treatment of DLBCL.

Artemisinin (ART) is one of the most potent and effective antimalarial molecules, first isolated from *Artemisia annua* L. in 1972 by Youyou Tu, who won the Nobel Prize in 2015. Since 1992, increasing number of studies have found that ART and its derivatives, such as dihydroartemisinin (DHA), artemether (ARM), and artesunate (ARS) have potent antitumor activities 17, 18, 19, 20. However, most of the ART derivatives, including ART itself, are insoluble or unstable in water, which significantly decreases the bioavailability of these drugs 21, 22. In order to overcome this shortcoming of ARTs, our group, together with Shanghai Institute of Materia Medica, brought specific structural modifications to ART and its derivatives. Among these modified products, SM1044, a new synthesized ART maleate, exhibited excellent water solubility, and the aqueous solution was quite stable.

Autophagy is an evolutionarily conserved process that degrades and eliminates the unwanted or dysfunctional intracellular components, such as misfolded proteins and organelles. Initiated by activation of the ULK1 complex, a double‐membrane vesicle called autophagosome is formed, which subsequently fuses with the lysosome, forming the autolysosome, whose inner contents are degraded and recycled. A number of autophagy‐related genes (ATGs) are involved in the regulation of autophagy, among which LC3/ATG8 plays a key role in the membrane formation of both the autophagosome and autolysosome 23. In most cases, autophagy protects cells from death under adverse conditions. Paradoxically, autophagy may also trigger cell death, including apoptosis 24, 25, 26, 27. However, the mechanism of autophagy‐dependent apoptosis has not yet been well elucidated.

In the present study, we observe a remarkable antitumor effect of SM1044 on several DLBCL cell lines and explore the possible mechanisms underlying the activity of SM1044. In short, we demonstrate that SM1044 treatment affects DLBCL cell survival both in vitro and in vivo, through the induction of autophagy as well as an autophagy‐dependent degradation of Survivin, followed by caspase‐dependent apoptosis.

## Materials and Methods

### Cell culture

DLBCL cell lines SU‐DHL‐4, SU‐DHL‐10, and OCI‐LY3 were obtained from the French National Institute of Health and Medical Research (INSERM). SU‐DHL‐4 was cultured in RPMI 1640 medium (Gibco, Carlsbad, CA) supplemented with 10% fetal bovine serum (FBS, Moregate Biotech, Bulimba, QLD, Australia). SU‐DHL‐10 was cultured in RPMI 1640 medium supplemented with 20% FBS. OCI‐LY3 was cultured in Iscove's modified Dulbecco's medium (Gibco) supplemented with 20% FBS. All three cell lines were cultured at 37°C in a 5% CO_2_ atmosphere. Authentication of cell line was performed and the profile was compared with that in DSMZ STR database.

### Reagents and antibodies

SM1044 was synthesized by Shanghai Institute of Materia Medica, Chinese Academy of Sciences, and dissolved in sterile purified water. ART, DHA, ARM, and ARS were generous gifts from Chongqing Huali Wulingshan Medicine company. Z‐VAD‐FMK and enhanced ATP assay kit were purchased from Beyotime Biotechnology (Haimen, Jiangsu, China). Chloroquine and bafilomycin A1 (Baf A1) were purchased from Sigma‐Aldrich (St. Louis, MO). MG132, STO‐609, l‐cycloserine, and cycloheximide (CHX) were purchased from Santa Cruz Biotechnology (Santa Cruz, CA). C646 and Compound C were purchased from Selleck Chemicals (Houston, TX). Sphingosine‐1‐phosphate (S1P) was obtained from LKT Laboratories (St. Paul, MN). Antibodies against caspase‐3, caspase‐8, caspase‐9, PARP, Survivin, XIAP, Flip, Bcl‐xL, Bcl‐w, A1/Bfl‐1, LC3, p‐AMPK, p‐mTOR, p‐ULK1, and p‐LKB1 were purchased from Cell Signaling Technology (CST, Danvers, MA); anti‐Bcl‐2, P300/CBP, and Mcl‐1 were purchased from Santa Cruz Biotechnology; *β*‐actin was purchased from Sigma‐Aldrich.

### Cell viability assay

Cell viability was determined by Cell Counting Kit‐8 (CCK8, Dojindo Laboratories, Kumamoto, Japan) according to the manufacturer's instructions. The percent inhibition was calculated using the following formula: Percent inhibition = [(Mean absorbance of the control well − Mean absorbance of the test well)/(Mean absorbance of the control well − Mean absorbance of the blank well)] × 100%.

### Apoptosis assessment by Annexin V staining

Cells were washed with 1× PBS buffer followed by 1× Annexin V buffer and resuspended in 200 *μ*L staining solution comprising 2 *μ*L fluorescein isothiocyanate‐Annexin V and 2 *μ*L propidiumiodide (PI) (FITC‐Annexin V staining kit, BD Biosciences, San Jose, CA). Then, 1 × 10^4^ cells were measured by flow cytometry. All data were collected and analyzed by the FlowJo software (FlowJo LLC, Ashland, Oregon, USA).

### Protein extraction, coimmunoprecipitation (co‐IP), and western blot analysis

Protein extraction, co‐IP, and western blot analysis were carried out as described previously 28.

### Transmission electron microscopy

SU‐DHL‐4 cells were fixed in 0.1 mol/L sodium cacodylate buffer (pH 7.3) containing 2% paraformaldehyde and 2.5% glutaraldehyde and embedded in resin mixture of Embed 812 and araldite. The samples were then dissected into 70‐nm‐thick slices and poststained with 3% uranyl acetate and Reynolds lead citrate. Finally, the samples were examined under FEI Tecnai Spirit G2 TEM and digital images were captured by an FEI Eagle camera.

### RNA extraction and real‐time PCR

Total RNA was extracted using Trizol reagent (Invitrogen, Carlsbad, CA) and the cDNA was obtained using an M‐MLV reverse transcriptase (Invitrogen). Real‐time quantitative polymerase chain reaction PCR (real‐time PCR) assay was performed using SuperReal PreMix Plus kit (Tiangen, Beijing, China). The primers were as follows: GAPDH (forward: GAAGGTGAAGGTCGGAGTC, reverse: GAAGATGGTGATGGGATTTC); Survivin (forward: TTTCTCAAGGACCACCGCATCTC, reverse: GCTCGTTCTCAGTGGGGCAGTG). Amplifications were performed in a 7500 Real‐Time PCR System (Applied Biosystems, Foster City, CA).

### Electroporation

Cells were centrifuged at 90*g* for 10 min, the supernatants were removed, the cell pellets were resuspended in 4D‐Nucleofector^™^ solution (SF cell line 4D‐Nucleofector X kit L, Lonza, Basel, Switzerland) containing the plasmids and transferred into the Nucleocuvette^™^ vessels. The vessels were then placed into the retainer of the 4D‐Nucleofector^™^ X unit and the Nucleofection^™^ process was ran with program DN‐100. After the run completed, the vessels were removed from the retainer and incubated for 10 min. The cells were resuspended with prewarmed medium and mixed by pipetting for three times, then plated onto cell culture plates for further experiments.

### Construction of lentiviral expression vectors

pLVX‐shRNA2 vector was obtained from Clontech Laboratories (Mountain View, CA). Recombinant lentiviral shLC3 (with a target sequence 5′‐CTGAGATCGATCAGTTCAT‐3′) was constructed according to the manufacturer's instructions. The pLVX‐IRES‐Puro vector was also obtained from Clontech Laboratories. The cDNA of Survivin was amplified by PCR and cloned into pLVX‐IRES‐Puro vector. The extended LC3‐interacting region (xLIR) motif mutated Survivin (forward primer: 5′‐GGTGAATTTTTGAAAGGGGACAGAGAA‐3′, reverse primer: 5′‐CCTTTCAAAAATTCACCAAGGGTTAAT‐3′) was constructed using a Fast Mutagenesis System (TransGen Biotechnology, Beijing, China).

### Enhanced ATP assay

The level of intracellular ATP was determined using an enhanced ATP assay kit (Beyotime Biotechnology) according to the manufacturer's instructions. Briefly, equal amounts of cells were harvested, lysed, and centrifuged at 12,000*g* for 5 min at 4°C. Then, 20 *μ*L of the supernatant was mixed with 100 *μ*L of luciferase reagent and the measurements were done using a microplate luminometer.

### Measurement of ceramide

Cell pellets were resuspended with 200 *μ*L 1× PBS, vortexed, and sonicated for 30 min. Then, 1.8 mL extraction solvent A (isopropanol/ethyl acetate, 1:2, v/v) was added, vortexed, and sonicated for another 30 min. After centrifugation at 2000*g* for 10 min, the supernatant was collected. Next, 2 mL extraction solvent B (isopropanol/ethyl acetate/H2O, 3:6:1, v/v/v) was added into the residue, vortexed, and sonicated for 30 min. After centrifugation, the supernatant was collected and mixed with the previous one. Then, the extraction was evaporated to dryness under nitrogen. The dried residue was dissolved in 500 *μ*L methanol, followed by filtration through a 0.45‐*μ*m PVDF membrane. Finally, 2 *μ*L solution was injected into a HPLC‐MS/MS system for test.

### In vivo xenograft model

Female nude mice (4‐ to 6‐week‐old) were housed and monitored at the Department of Laboratory Animal Science, Shanghai Jiao Tong University School of Medicine. All animal experiments were approved by the Institutional Review Board of Shanghai Jiao Tong University School of Medicine, and complied with the ARRIVE guidelines. Nude mice were subcutaneously inoculated in the right flank with 1.5 × 10^7^ SU‐DHL‐4 cells suspended in 100 *μ*L 1× PBS. The two largest perpendicular axes were measured with standard calipers, and tumor volume was calculated using the following formula: (length) × (width)^2^/2 as previously reported 28. When tumors reached 5 mm in the length, mice were randomly separated into vehicle control (normal saline, *n* = 11) or SM1044 (5 mg/kg per day, *n* = 12) treatment groups. Tumor size and body weight were measured, and mice were photographed every 2 days. Animals were euthanized when tumors reached 15 mm in the length.

### Statistical analyses

The data are expressed as the mean ± SEM. The significance was calculated using Student's *t*‐test. A value of *P* < 0.05 was considered to be significant.

## Results

### Treatment of DLBCL cell lines with SM1044 inhibits cell growth and induces apoptosis

**Figure 1:**
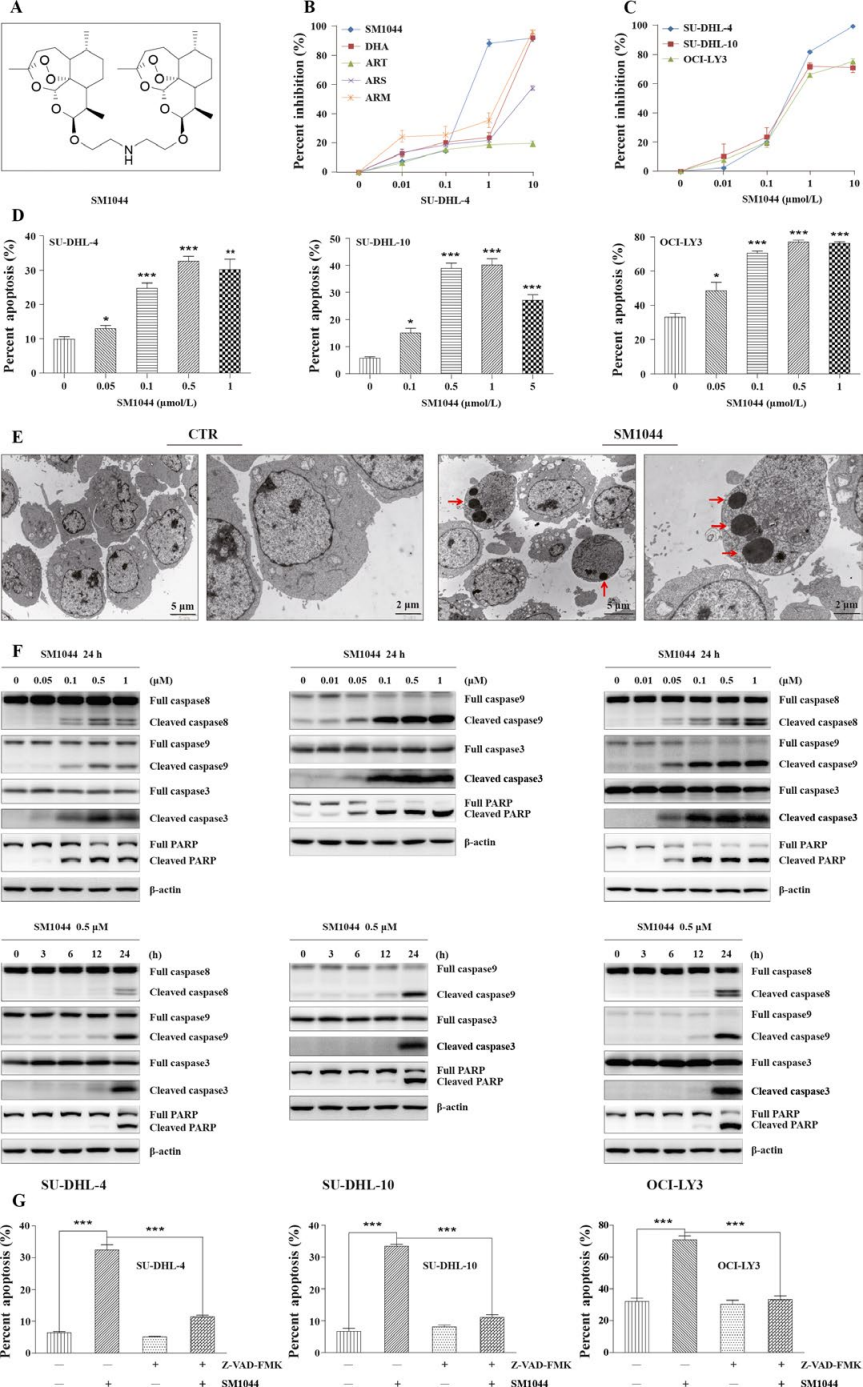
SM1044 induces caspase‐dependent apoptosis in DLBCL cell lines. (A) Structural formula of SM1044. (B) SU‐DHL‐4 cells were treated with the indicated concentrations of SM1044, DHA, ART, ARS, and ARM for 24 h, respectively. Cell viability was measured by CCK‐8 and the percent inhibitions were calculated (mean ± SEM, *n* = 3). (C) SU‐DHL‐4, SU‐DHL‐10, and OCI‐LY3 cells were treated with the indicated concentrations of SM1044 for 24 h. The cell viability was measured by CCK‐8 and the percent inhibitions were calculated (mean ± SEM, *n* = 3). (D) SU‐DHL‐4, SU‐DHL‐10, and OCI‐LY3 cells were treated with the indicated concentrations of SM1044 for 24 h. Cell apoptosis was detected by flow cytometry using the FITC‐Annexin V/PI apoptosis detection kit (mean ± SEM, *n* = 3). SU‐DHL‐4: 0 *μ*mol/L versus 0.05 *μ*mol/L, *P* = 0.042; 0.1 *μ*mol/L, *P* < 0.001; 0.5 *μ*mol/L, *P* < 0.001; 1 *μ*mol/L, *P* = 0.0061. SU‐DHL‐10: 0 *μ*mol/L versus 0.1 *μ*mol/L, *P* = 0.023; 0.5 *μ*mol/L, *P* < 0.001; 1 *μ*mol/L, *P* < 0.001; 5 *μ*mol/L, *P* < 0.001. OCI‐LY3: 0 *μ*mol/L versus 0.05 *μ*mol/L, *P* = 0.034; 0.1 *μ*mol/L, *P* < 0.001; 0.5 *μ*mol/L, *P* < 0.001; 1 *μ*mol/L, *P* < 0.001. (E) SU‐DHL‐4 cells were treated with SM1044 for 24 h. Representative electron microscopy photomicrographs are shown. The red arrows point to the apoptosis bodies. (F) The expressions of caspase‐8, ‐9, ‐3, and PARP were detected by western blot in SU‐DHL‐4, SU‐DHL‐10, and OCI‐LY3 cells treated with the indicated concentrations of SM1044 for the indicated time courses. (G) Apoptosis was measured in SU‐DHL‐4, SU‐DHL‐10, and OCI‐LY3 cells pretreated with caspase inhibitor Z‐VAD‐FMK for 1 h, and then treated with SM1044 for another 24 h (mean ± SEM, *n* = 3). SU‐DHL‐4: SM1044 versus control, *P* < 0.001; SM1044 versus Z‐VAD‐FMK plus SM1044, *P* < 0.001. SU‐DHL‐10: SM1044 versus control, *P* < 0.001; SM1044 versus Z‐VAD‐FMK plus SM1044, *P* < 0.001. OCI‐LY3: SM1044 versus control, *P* < 0.001; SM1044 versus Z‐VAD‐FMK plus SM1044, *P* < 0.001. *P < 0.05, **P < 0.01, ***P < 0.001.

First, we evaluated the effects of a SM1044 (Fig. 1A) treatment on cell survival in several lymphoma cell lines and found that SM1044 severely affected cell viability, especially in SU‐DHL‐4, a typical DLBCL cell line. Then, we compared the respective effects of different ART derivatives, which identified SM1044 as the most effective one (Fig. 1B). Three DLBCL cell lines, SU‐DHL‐4, SU‐DHL‐10, and OCI‐LY3 were then used to test the effectiveness of SM1044 on cell viability, with the findings that all three cell lines were highly sensitive to SM1044 (Fig. 1C).

We then observed that SM1044 treatment increased the proportion of cells in the G0/G1 phase of the cell cycle while decreasing the number of cells in S phase, suggesting that SM1044 could cause a G1 phase arrest (Fig. S1A). Apoptosis, tested using the FITC‐Annexin V/PI method, was also found to be significantly induced by SM1044 (*SU‐DHL‐4*: 0 *μ*mol/L vs. 0.05 *μ*mol/L, *P* = 0.042; 0.1 *μ*mol/L, *P* < 0.001; 0.5 *μ*mol/L, *P* < 0.001; 1 *μ*mol/L, *P* = 0.0061; *SU‐DHL‐10*: 0 *μ*mol/L vs. 0.1 *μ*mol/L, *P* = 0.023; 0.5 *μ*mol/L, *P* < 0.001; 1 *μ*mol/L, *P* < 0.001; 5 *μ*mol/L, *P* < 0.001; *OCI‐LY3*: 0 *μ*mol/L vs. 0.05 *μ*mol/L, *P* = 0.034; 0.1 *μ*mol/L, *P* < 0.001; 0.5 *μ*mol/L, *P* < 0.001; 1 *μ*mol/L, *P* < 0.001) (Fig. 1D). Likewise, SM1044 also induced a loss of mitochondrial membrane potential (MMP) (0 *μ*mol/L vs. 0.05 *μ*mol/L, *P* = 0.0017; 0.1 *μ*mol/L, *P* = 0.0070; 0.5 *μ*mol/L, *P* < 0.001; 1 *μ*mol/L, *P* < 0.001) (Fig. S1B). Furthermore, the appearance of apoptotic bodies was also observed by transmission electron microscopy (TEM) (Fig. 1E), as well as the cleavage of apoptosis‐related proteins caspase‐8, ‐9, ‐3, and PARP by western blot (Fig. 1F). It is of note that, in agreement with a previous report 29, we observed that caspase‐8 was rarely expressed in SU‐DHL‐10 cell line (data not shown). Moreover, apoptosis induced by SM1044 was significantly reduced by a pretreatment with the pan‐caspase inhibitor Z‐VAD‐FMK, demonstrating that the apoptosis triggered by SM1044 was caspase‐dependent (*SU‐DHL‐4*: SM1044 vs. control, *P* < 0.001; SM1044 vs. Z‐VAD‐FMK plus SM1044, *P* < 0.001; *SU‐DHL‐10*: SM1044 vs. control, *P* < 0.001; SM1044 vs. Z‐VAD‐FMK plus SM1044, *P* < 0.001; *OCI‐LY3*: SM1044 vs. control, *P* < 0.001; SM1044 vs. Z‐VAD‐FMK plus SM1044, *P* < 0.001) (Fig. 1G).

### SM1044 induces autophagy in DLBCL cell lines

**Figure 2:**
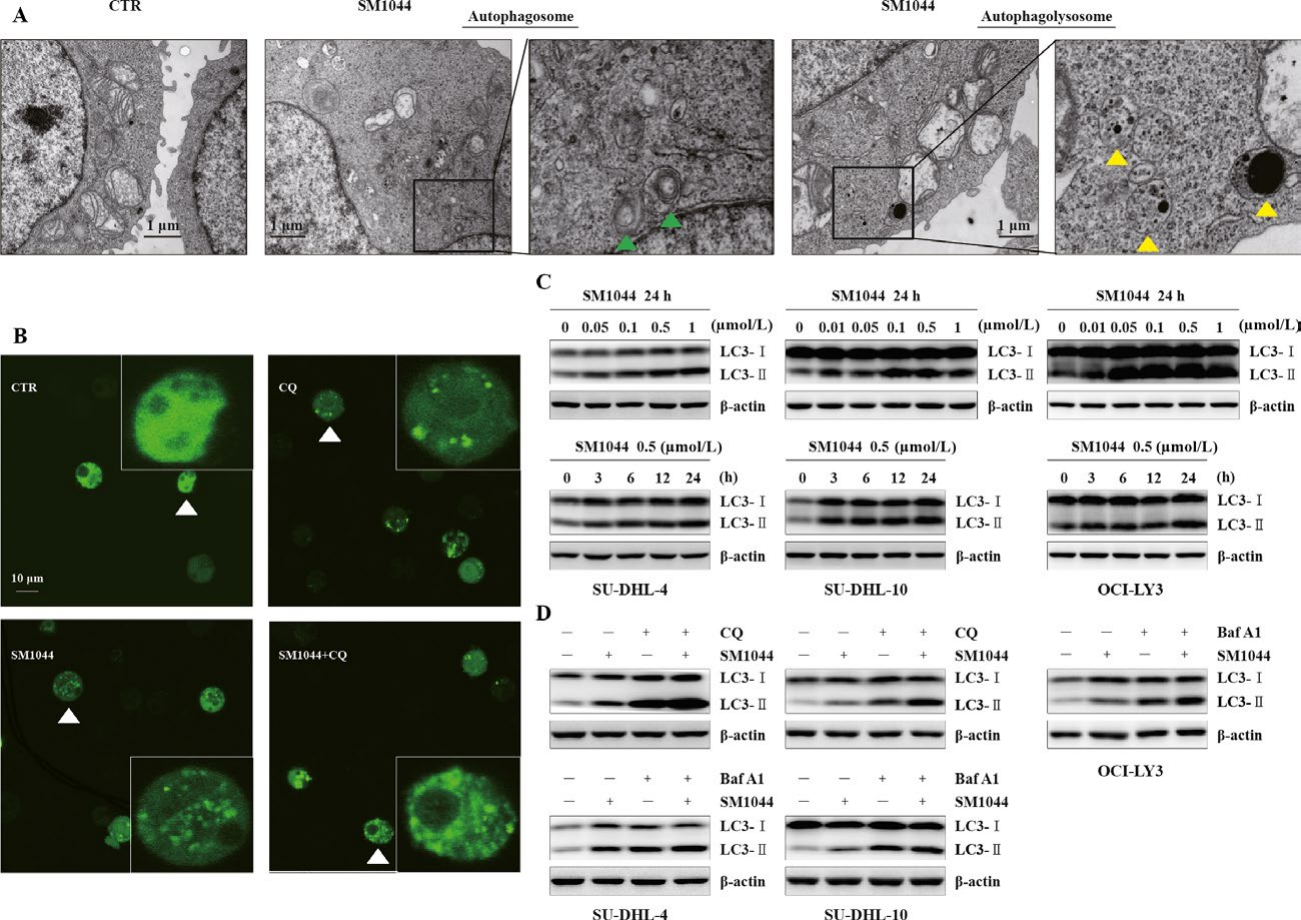
SM1044‐induced autophagy. (A) SU‐DHL‐4 cells were treated with SM1044 for 24 h. Representative electron microscopy photomicrographs are shown. The green triangles indicate autophagosomes and the yellow triangles indicate autophagolysosomes. (B) GFP‐LC3 plasmids were transfected into SU‐DHL‐4 cells. Forty‐eight hours later, cells were pretreated by CQ for 1 h, followed by a SM1044 treatment for another 24 h. LC3 puncta are shown by fluorescence microscope. (C) SU‐DHL‐4, SU‐DHL‐10, and OCI‐LY3 cells were treated with the indicated concentrations of SM1044 for the indicated time courses. The expression of LC3 was detected by western blot. (D) SU‐DHL‐4, SU‐DHL‐10, and OCI‐LY3 cells were pretreated with CQ or Baf A1 for 1 h, followed by a SM1044 treatment for another 24 h. The expression of LC3 was detected by western blot.

While observing apoptotic bodies by TEM, we also detected the appearance of typical autophagic compartments in SM1044‐treated cells in contrast to untreated cells (Fig. 2A). Then, in order to investigate the contribution of the autophagy‐related gene LC3/ATG8 pathway, GFP‐LC3 was transiently transfected into SU‐DHL‐4 cells by electroporation. The punctuate GFP‐LC3 fluorescence was induced by SM1044, and was further increased after cells were pretreated with the autophagy inhibitor CQ and then by SM1044 (Fig. 2B). Besides, the levels of LC3‐II increased in a concentration‐ and time‐dependent manner, and were further augmented after the pretreatment by CQ or Baf A1 (another inhibitor of autophagy) (Fig. 2C and D). It is worth mentioning that, since CQ itself induced a significant apoptosis in OCI‐LY3 cells, data of CQ group in OCI‐LY3 cell line are not shown here or below.

Taken together, these findings support the hypothesis that SM1044 is a potent inducer of autophagy.

### SM1044‐induced apoptosis is autophagy dependent

**Figure 3:**
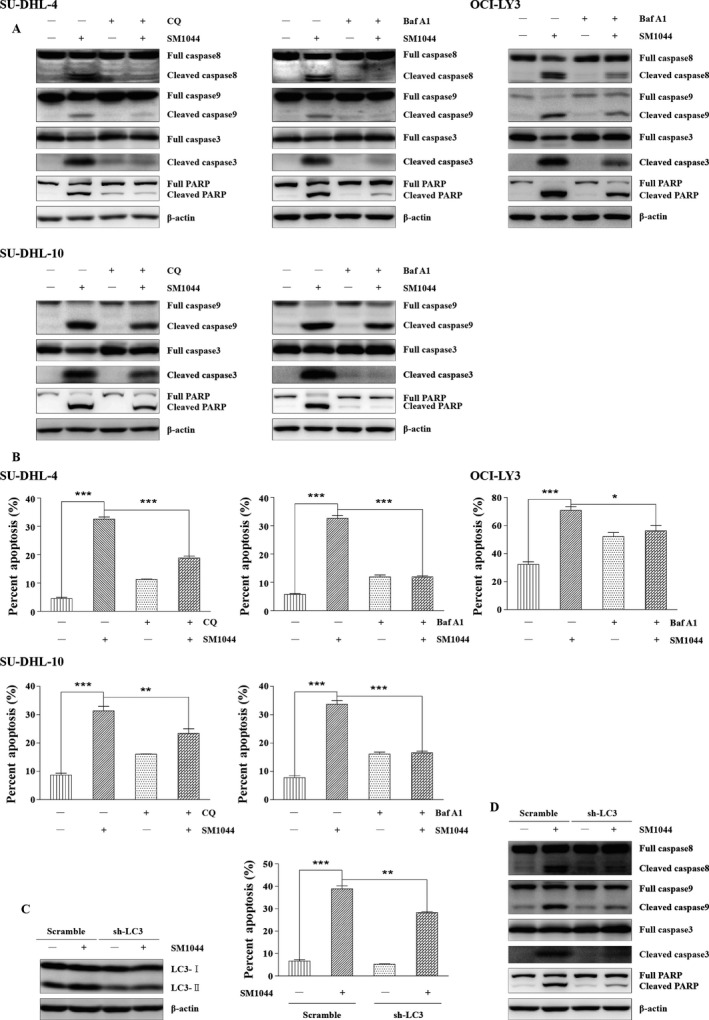
SM1044‐induced apoptosis is dependent on the generation of autophagy. (A) SU‐DHL‐4, SU‐DHL‐10, and OCI‐LY3 cells were pretreated with CQ or Baf A1 for 1 h, followed by SM1044 treatment for another 24 h. The respective expressions of caspase‐8, ‐9, ‐3, and PARP were detected by western blot. (B) Apoptosis was measured by flow cytometry (mean ± SEM, *n* = 3). SU‐DHL‐4: SM1044 versus control, *P* < 0.001; SM1044 versus CQ plus SM1044, *P* < 0.001; SM1044 versus Baf A1, *P* < 0.001. SU‐DHL‐10: SM1044 versus control, *P* < 0.001; SM1044 versus CQ plus SM1044, *P* = 0.0077; SM1044 versus Baf A1, *P* < 0.001. OCI‐LY3: SM1044 versus control, *P* < 0.001; SM1044 versus Baf A1, *P* = 0.041. (C) SU‐DHL‐4 cells were stably transfected with scramble or LC3 shRNA, then treated with SM1044 for 24 h. Apoptosis was measured by flow cytometry (mean ± SEM, *n* = 3). Scramble + SM1044 versus sh‐LC3 + SM1044, *P* = 0.0012. (D) The respective expressions of caspase‐8, ‐9, ‐3, and PARP were detected by western blot in the stably transfected cells after SM1044 treatment for 24 h. *P < 0.05, **P < 0.01, ***P < 0.001.

As shown above, both apoptosis and autophagy were detected in SU‐DHL‐4 cells following the addition of SM1044. Interestingly, our western blot results suggested that autophagy was induced at much lower concentrations and at much earlier time course compared to apoptosis (Figs. 1F and 2C). In agreement with this observation, TEM showed that, although both autophagy compartments and apoptotic bodies were induced 24 h after the addition of 0.5 *μ*mol/L SM1044, only autophagy compartments were found 6 h after the addition of 0.01 *μ*mol/L SM1044 (Fig. S2). Strikingly, the cleavage of caspase‐8, ‐9, ‐3, and PARP was reduced, apoptosis was inhibited, and cell viability was increased after the pretreatment of cells with CQ or Baf A1, indicating that cells were protected from apoptosis when autophagy was inhibited (*SU‐DHL‐4*: SM1044 vs. control, *P* < 0.001; SM1044 vs. CQ plus SM1044, *P* < 0.001; SM1044 vs. Baf A1, *P* < 0.001; *SU‐DHL‐10*: SM1044 vs. control, *P* < 0.001; SM1044 vs. CQ plus SM1044, *P* = 0.0077; SM1044 vs. Baf A1, *P* < 0.001; *OCI‐LY3*: SM1044 vs. control, *P* < 0.001; SM1044 vs. Baf A1, *P* = 0.041) (Figs. 3A and B, S3). Therefore, SM1044‐induced apoptosis appeared to be autophagy dependent. In order to further test this hypothesis, the expression of LC3 in SU‐DHL‐4 cells was stably knocked down after the expression of a specific anti‐LC3 shRNA. As expected, cell apoptosis was reduced in the LC3 knockdown cells compared to the control cells (Scramble + SM1044 vs. sh‐LC3 + SM1044, *P* = 0.0012) (Fig. 3C and D). Collectively, these findings demonstrate that apoptosis induced by SM1044 is autophagy dependent.

### SM1044 treatment induces an autophagy‐dependent degradation of Survivin

**Figure 4:**
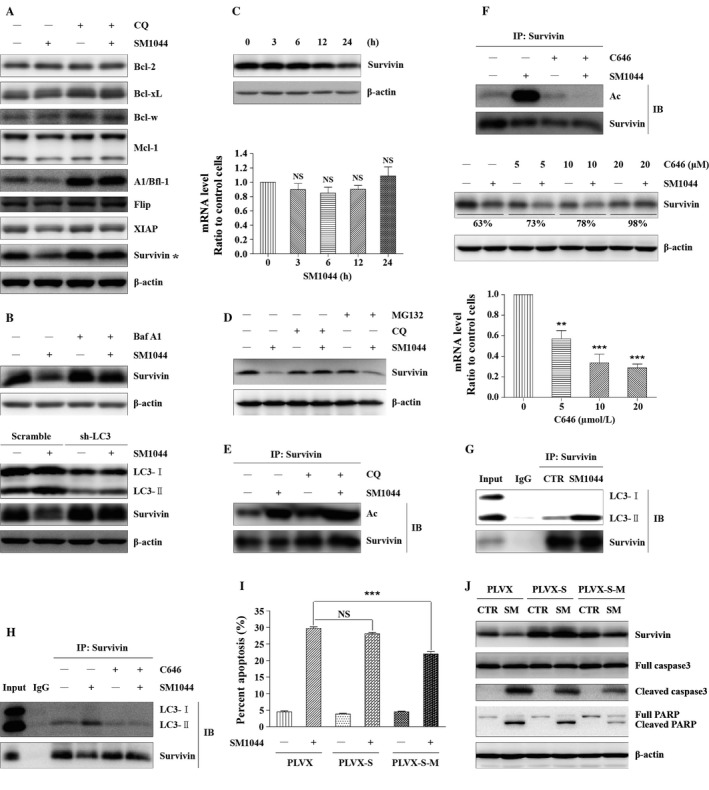
SM044 promotes the acetylation of Survivin, enhances the interaction between Survivin and LC3‐II, leading to the degradation of Survivin and apoptosis. (A) SU‐DHL‐4 cells were pretreated with CQ for 1 h, followed by SM1044 treatment for another 24 h. The respective expressions of antiapoptosis proteins were detected by western blot. (B) SU‐DHL‐4 cells were pretreated with Baf A1 for 1 h, followed by SM1044 treatment for another 24 h. The expression of Survivin was detected by western blot (upper panel). Cells with a stable knockdown of LC3 or control cells (scramble shRNA) were treated with SM1044 for 24 h. The expression of Survivin was detected by western blot (lower panel). (C) SU‐DHL‐4 cells were treated with SM1044 for the indicated time courses. The protein and mRNA levels of Survivin were, respectively, detected by western blot (upper panel) and real‐time PCR (lower panel, mean ± SEM, *n* = 3). (D) SU‐DHL‐4 cells were pretreated with MG132 or CQ for 1 h, followed by a SM1044 treatment for another 24 h. The expression of Survivin was detected by western blot. (E) SU‐DHL‐4 cells were pretreated with CQ for 1 h, followed by SM1044 treatment for another 12 h. Survivin was immunoprecipitated with its antibody. Acetylation of Survivin was detected by western blot. (F) SU‐DHL‐4 cells were pretreated with the p300 inhibitor C646 for 1 h, followed by SM1044 treatment for another 24 h. Acetylation of Survivin was detected by western blot after Survivin was immunoprecipitated with its antibody (upper panel). The protein and mRNA levels of Survivin were detected by western blot (middle panel) and Real‐time PCR (lower panel, mean ± SEM, *n* = 3), respectively. For real‐time PCR, C646: 0 versus 5 *μ*mol/L, *P* = 0.0086; 10 *μ*mol/L, *P* < 0.001; 20 *μ*mol/L, *P* < 0.001. The ratio of Survivin (with/without SM1044) at the protein level in different concentration groups of C646 was calculated using ImageJ software. (G) SU‐DHL‐4 cells were treated with SM1044 for 12 h. Cell lysates were immunoprecipitated using anti‐Survivin antibody (IP: Survivin) or normal rabbit IgG. The respective levels of Survivin and LC3 were detected by western blot. (H) SU‐DHL‐4 cells were pretreated with C646 for 1 h, followed by SM1044 treatment for another 12 h. Cell lysates were immunoprecipitated using the anti‐Survivin antibody. The levels of Survivin and LC3 were detected by western blot. (I) SU‐DHL‐4 cells were stably transfected with empty vector, Survivin, or xLIR motif mutated Survivin (PLVX, PLVX‐S, PLVX‐S‐M), respectively. The stable transfected cells were treated with SM1044 for 24 h. Apoptosis was measured by flow cytometry (mean ± SEM, *n* = 3). PLVX + SM1044 versus PLVX‐S‐M + SM1044, *P* < 0.001. (J) The stable transfected cells were treated with SM1044 (SM) for 24 h. The levels of Survivin, caspase‐3 and PARP were detected by western blot. **P < 0.01, ***P < 0.001 compared with the control group without C646 treatment. NS, no significance.

We next studied the molecular mechanism of autophagy‐dependent apoptosis. As autophagy is also recognized as a protein degradation system, we speculated that autophagy might cause apoptosis through degrading one or several antiapoptosis proteins. Our hypothesis was that these antiapoptosis proteins could be degraded by SM1044 treatment while being preserved by the pretreatment of autophagy inhibitors. To search for this/these proteins, SU‐DHL‐4 cells were divided into four groups: (1) control group, treated with solvent for 24 h; (2) SM1044 group, treated with 0.5 *μ*mol/L SM1044 for 24 h; (3) CQ group, treated with 100 *μ*mol/L CQ for 25 h; (4) SM1044 + CQ group, pretreated with 100 *μ*mol/L CQ for 1 h, then adding 0.5 *μ*mol/L SM1044 for another 24 h. Among several candidate antiapoptotic factors, Survivin appeared to be the best target for the SM1044‐induced degradation by autophagy (Fig. 4A). The autophagic degradation of Survivin was further confirmed by the pretreatment of cells with Baf A1 and by the knockdown of LC3 (sh‐LC3) (Fig. 4B). Subsequently, we found that Survivin starts decreasing 12 h after the addition of SM1044, which is earlier than cell apoptosis but later than the induction of autophagy (Fig. 4C). Notably, SM1044 decreased the protein level of Survivin, but not its mRNA level (Fig. 4C). Meanwhile, we found that a treatment by CQ prevented the degradation of Survivin, whereas the proteasome inhibitor MG132 did not stabilize the protein, suggesting that the degradation of Survivin depended on autophagy, rather than on the proteasome (Fig. 4D).

### SM1044 treatment stimulates Survivin acetylation by p300/CBP, which is required for its autophagy‐dependent degradation

Then, we wanted to know why Survivin was degraded by autophagy after the addition of SM1044. It is reported that acetylated Survivin is less stable than its deacetylated form 30. Our results showed that the acetylation level of Survivin was significantly enhanced by SM1044 and was further increased by the pretreatment by CQ (Fig. 4E). Acetylation is triggered and regulated by a series of acetyltransferases, among which, p300/CBP is one of the most important ones. Thus, C646 (a p300/CBP inhibitor) was used to inhibit the acetyltransferase activity of p300/CBP. As a result, we found that C646 significantly reversed the acetylation of Survivin induced by SM1044. More importantly, although C646 downregulated the expression of Survivin both at its mRNA (C646: 0 *μ*mol/L vs. 5 *μ*mol/L, *P* = 0.0086; 10 *μ*mol/L, *P* < 0.001; 20 *μ*mol/L, *P* < 0.001) and protein levels, the ratio of Survivin (with/without SM1044) at the protein level was increased in a concentration‐dependent manner (63%, 73%, 78%, 98%), indicating that C646 was able to inhibit the turnover of Survivin (Fig. 4F). Taken together, these findings indicate that Survivin is acetylated by p300/CBP and thus degraded through autophagy after the SM1044 treatment.

### SM1044 induces the interaction of Survivin and LC3‐II, leading to the degradation of Survivin and the induction of apoptosis

To clarify how acetylated Survivin can be degraded by autophagy, we analyzed the amino acid sequence of Survivin and found an extended LC3‐interacting region motif (xLIR motif) “EFLKL,” which is highly conserved during evolution. Hence, we tested the possibility of an interaction between LC3‐II and Survivin in SU‐DHL‐4 cells. Our results showed that Survivin slightly binds to LC3‐II in the absence of SM1044, and that this binding significantly increases after the addition of SM1044 (Fig. 4G). Interestingly, we also observed that C646 abolishes the enhanced interaction between Survivin and LC3‐II, suggesting that the interaction between these two proteins is dependent on the acetylation of Survivin (Fig. 4H).

In order to further investigate the role of Survivin degradation in the induction of apoptosis, PLVX‐IRES‐Puro vector (PLVX), Survivin (PLVX‐S), and xLIR motif mutated Survivin (PLVX‐S‐M) were, respectively, stably transfected into SU‐DHL‐4 cells. While the overexpression of Survivin showed weak ability to decrease the SM1044‐induced apoptosis, the overexpression of PLVX‐S‐M significantly rescued SU‐DHL‐4 cells from apoptosis (PLVX + SM1044 vs. PLVX‐S‐M + SM1044, *P* < 0.001) (Fig. 4I). In accordance with these results, cleaved caspase‐3 and PARP were reduced in the PLVX‐S‐M overexpression experimental condition compared to the vector condition (Fig. 4J).

### SM1044 induces CaMKK2–AMPK–ULK1 axis‐mediated autophagy through promoting de novo synthesis of ceramide

**Figure 5:**
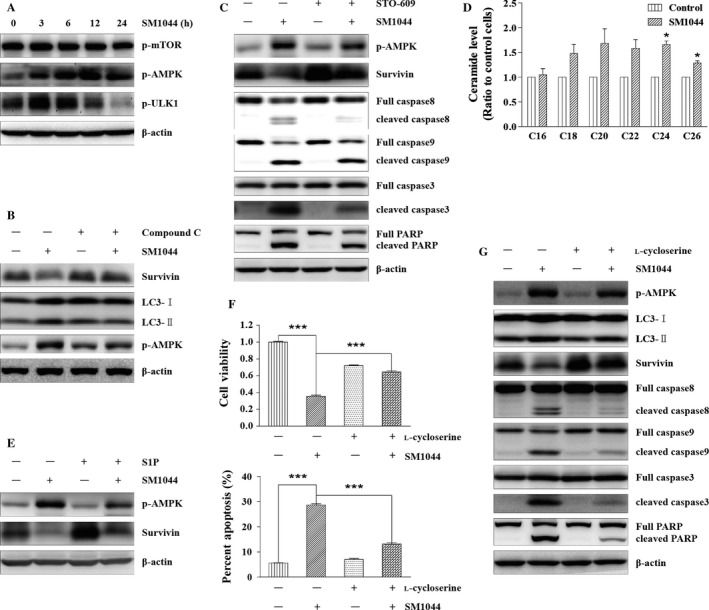
SM1044 induces CaMKK2–AMPK–ULK1 pathway‐mediated autophagy through promoting de novo synthesis of ceramide. (A) SU‐DHL‐4 cells were treated with SM1044 for the indicated time courses. The active ULK1, AMPK and mTOR were detected by western blot. (B) SU‐DHL‐4 cells were pretreated with AMPK inhibitor compound C for 1 h, followed by SM1044 treatment for another 24 h. The levels of LC3, Survivin, and p‐AMPK were detected by western blot. (C) SU‐DHL‐4 cells were pretreated with CaMKK2 inhibitor STO‐609 for 1 h, followed by SM1044 treatment for another 24 h. The levels of p‐AMPK, Survivin, caspase‐8, ‐9, ‐3, and PARP were detected by western blot. (D) SU‐DHL‐4 cells were treated with SM1044 for 3 h and the level of ceramide was detected by HPLC‐MS/MS. C24: Control versus SM1044, *P* = 0.011. C26: Control versus SM1044, *P* = 0.023. (E) SU‐DHL‐4 cells were pretreated with ceramide inhibitor S1P for 1 h, followed by SM1044 treatment for another 24 h. The levels of p‐AMPK and Survivin were detected by western blot. (F) SU‐DHL‐4 cells were pretreated with ceramide de novo synthesis inhibitor l‐cycloserine for 1 h, followed by SM1044 treatment for another 24 h. Cell viability was detected by CCK‐8 (upper panel, mean ± SEM, *n* = 3) and apoptosis was measured by flow cytometry (lower panel, mean ± SEM, *n* = 3). Cell viability: SM1044 versus control, *P* < 0.001, SM1044 versus l‐cycloserine plus SM1044, *P* < 0.001. Percent apoptosis: SM1044 versus control, *P* < 0.001, SM1044 versus l‐cycloserine plus SM1044, *P* < 0.001. (G) SU‐DHL‐4 cells were pretreated with l‐cycloserine for 1 h, followed by SM1044 treatment for another 24 h. The levels of p‐AMPK, LC3, Survivin, caspase‐8, ‐9, ‐3, and PARP were detected by western blot. *P < 0.05, ***P < 0.001.

To further investigate how autophagy was induced by SM1044, we monitored the activation of ULK1, one of the most important autophagy initiation factors, along with its upstream regulators mTOR and AMPK. ULK1 and AMPK were activated as early as 3 h after the addition of SM1044, while mTOR was not (Fig. 5A). Then, compound C (an AMPK inhibitor) was used to inhibit the activation of AMPK, resulting in a reduced level of LC3‐II and a rescue of the expression of Survivin (Fig. 5B). Collectively, these observations suggested that the activation of AMPK is the main cause of SM1044‐induced autophagy and autophagy‐dependent degradation of Survivin.

AMPK is activated by LKB1 under the condition of an elevated AMP/ATP (or ADP/ATP) ratio 31. We observed that LKB1 was persistently activated with or without the addition of SM1044 (Fig. S4A). However, we found no significant change in the cellular ATP level after the addition of SM1044, indicating that AMPK is not activated by LKB1 in our model system (Fig. S4B). AMPK has also been reported to be activated by Ca^2+^/calmodulin‐dependent kinase kinases (CaMKKs), especially CaMKK2, which is independent from changes in adenine nucleotides 32. Therefore, we tested the effect of STO‐609, an inhibitor of CaMKK2. As a result, we observed that STO‐609 reversed the activation of AMPK induced by SM1044 and rescued the expression of Survivin. Accordingly, the respective cleavages of caspase‐8, ‐9, ‐3, and PARP were also reduced by STO‐609 (Fig. 5C). Taken together, these results demonstrate that AMPK is activated by CaMKK2 after the treatment of SM1044.

Tetrahydrocannabinol (THC) is reported to induce the synthesis of ceramide, thus mediating autophagy‐dependent apoptosis through CaMKK2 33. It is also reported that ART is able to induce the generation of ceramide 34. Thus, we detected ceramide using high‐performance liquid chromatography–mass spectrometry (HPLC‐MS/MS) and found increased levels of C24 and C26 ceramide 3 h after the addition of SM1044 (C24: control vs. SM1044, *P* = 0.011; C26: control vs. SM1044, *P* = 0.023). Besides, C18, C20, and C22 ceramides also had a tendency to increase (*P* > 0.05) (Fig. 5D). Next, S1P (an inhibitor of ceramide) was used to interfere with ceramide signaling showing a diminished activation of AMPK compared to the solvent control group after SM1044 treatment (Fig. 5E). Interestingly, the expression of Survivin was also rescued by S1P (Fig. 5E). Moreover, we observed that l‐cycloserine, the specific inhibitor of de novo synthesis of ceramide, could reverse SM1044‐induced cell viability inhibition and apoptosis (*cell viability*: SM1044 vs. control, *P* < 0.001, SM1044 vs. l‐cycloserine plus SM1044, *P* < 0.001; *percent apoptosis*: SM1044 vs. control, *P* < 0.001, SM1044 vs. l‐cycloserine plus SM1044, *P* < 0.001) (Fig. 5F). Furthermore, we found that l‐cycloserine also inhibited the activation of AMPK, the formation of LC3‐II, the degradation of Survivin, as well as the cleavages of caspase‐8, ‐9, ‐3, and PARP (Fig. 5G). Taken together, these results demonstrate that SM1044 can induce the de novo synthesis of ceramide and thus activate AMPK through CaMKK2.

### ARTs degrade Survivin in DLBCL cell lines

**Figure 6:**
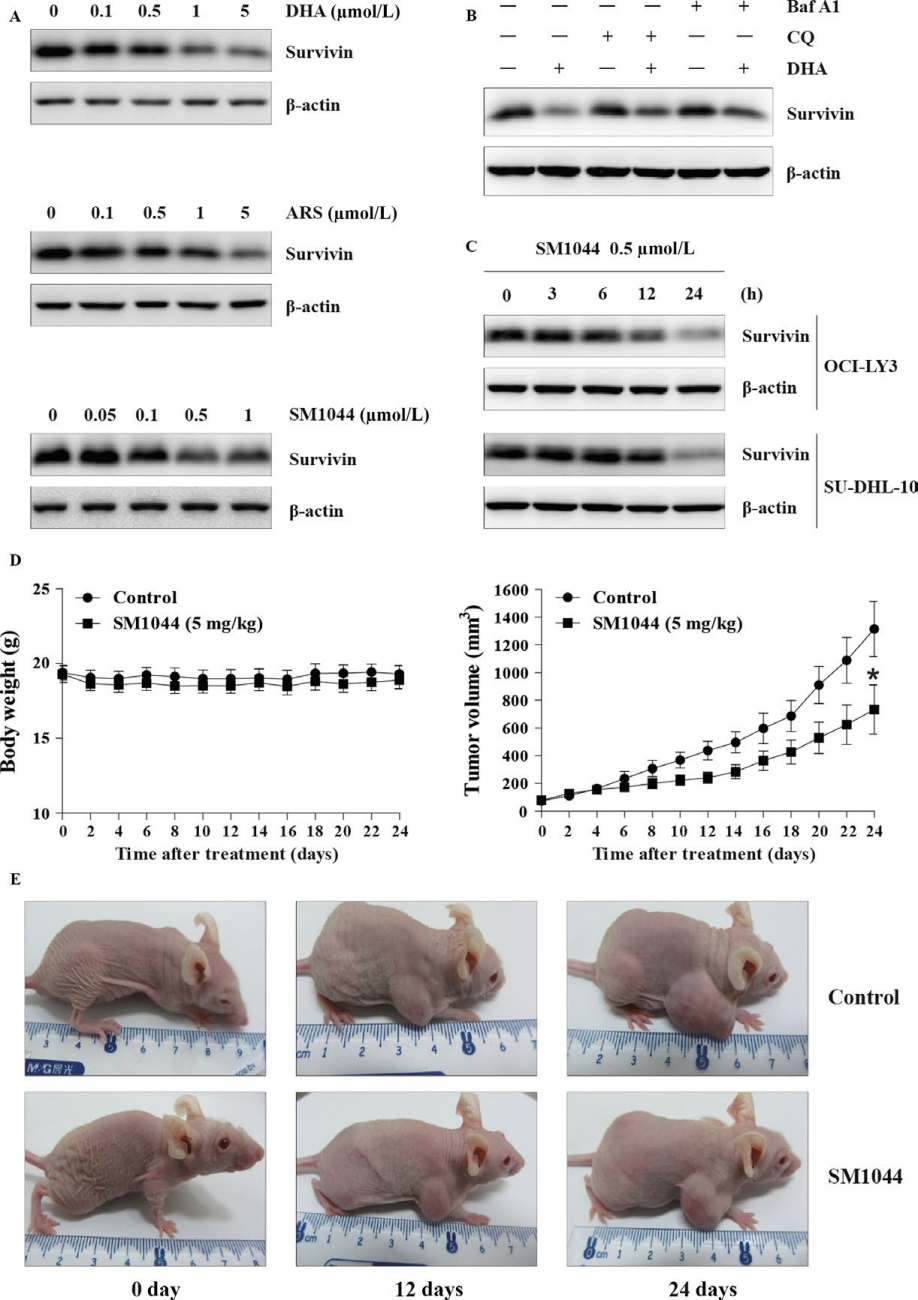
Degradation of Survivin by ARTs and in vivo effect of SM1044. (A) SU‐DHL‐4 cells were treated with ART and several ART derivatives (as indicated). The expression of Survivin was detected by western blot. (B) SU‐DHL‐4 cells were pretreated with CQ or Baf A1 for 1 h, followed by DHA treatment for another 24 h. The expression of Survivin was detected by western blot. (C) SU‐DHL‐10 and OCI‐LY3 cells were treated with SM1044 for the indicated time courses. The expression of Survivin was detected by western blot. (D) Nude mice with grafted tumors were treated with SM1044 (*n* = 12, 5 mg/kg per day) or vehicle control (*n* = 11, control) every day. Body weights and tumor volumes were measured every other day and plotted on the curve diagram. On day 0, the mean body weights of normal saline‐treated and SM1044‐treated groups were (19.4 ± 0.5) g and (19.3 ± 0.6) g (*P* = 0.89), respectively, and the mean tumor volumes were (73.6 ± 13.6) mm^3^ and (77.6 ± 14.2) mm^3^ (*P* = 0.84), respectively. However, on day 24, the mean body weights of normal saline‐treated and SM1044‐treated groups were (19.3 ± 0.5) g and (18.9 ± 0.6) g (*P* = 0.60), respectively, while the mean tumor volumes were (1315.2 ± 200.4) mm^3^ and (734.3 ± 177.4) mm^3^ (*P* = 0.04), respectively. *P < 0.05. (E) Representative images of control and SM1044‐treated tumors.

Considering that SM1044 is an ART derivative, we further assessed the effects of other ARTs on the degradation of Survivin in SU‐DHL‐4 cells. The results showed that besides SM1044, DHA and ARS could also downregulate the expression of Survivin (Fig. 6A). We also found that both CQ and Baf A1 could reverse the degradation of Survivin induced by DHA (Fig. 6B). Interestingly, we also found a time‐dependent degradation of Survivin by SM1044 in both OCI‐LY3 and SU‐DHL‐10, the other two DLBCL cell lines (Fig. 6C).

### The in vivo effects of SM1044 on DLBCL

Finally, we tested the effects of SM1044 in vivo. Nude mice models of human DLBCL were established by subcutaneously inoculating SU‐DHL‐4 cells. Tumor‐bearing mice were treated with normal saline (*n* = 11) or SM1044 (*n* = 12), respectively, and the body weight and the tumor volume of each mouse was measured every other day. Although there was no difference in tumor sizes before SM1044 treatment, the growth rate was lower in the SM1044‐treated group compared to the normal saline‐treated group (Fig. 6D and E). On day 0, the mean body weights of normal saline‐treated and SM1044‐treated groups were (19.4 ± 0.5) g and (19.3 ± 0.6) g (*P* = 0.89), respectively, and the mean tumor volumes were (73.6 ± 13.6) mm^3^ and (77.6 ± 14.2) mm^3^ (*P* = 0.84), respectively. However, on day 24, the mean body weights of normal saline‐treated and SM1044‐treated group were (19.3 ± 0.5) g and (18.9 ± 0.6) g (*P* = 0.60), respectively, while the mean tumor volumes were (1315.2 ± 200.4) mm^3^ and (734.3 ± 177.4) mm^3^ (*P* = 0.04), respectively.

## Discussion

Although nowadays with the R‐CHOP regimen more than half of DLBCL patients are able to achieve sustained remission, the outcome of nonresponders is still dismal. Therefore, additional effective and well‐tolerated drugs are needed. The antitumor activity of ART, a safe and highly effective antimalarial drug, has been demonstrated both in vitro and in animal models 18, 19. Better yet, ARS, a water‐soluble derivative of ART, is proved to be effective for DLBCL treatment, and has also been reported to sensitize B‐cell lymphoma to rituximab 20, 21. To further improve the anti‐DLBCL effect of ARTs, we synthesized SM1044, a new water‐soluble ART maleate, and demonstrated that, among several tested ARTs, SM1044 has the best inhibitory effect on the DLBCL cell line SU‐DHL‐4. We also observed that SM1044 induces apoptosis in DLBCL cell lines. More interestingly, we found that the apoptosis induced by SM1044 is dependent on autophagy induction.

As a “self‐eating” process, autophagy acts as a double‐edged sword. On the one hand, through degrading and recycling cytoplasm and organelles, autophagy generates energy and materials to produce new proteins or membranes, allowing cells to adapt to the adverse environment, thus protecting cells from death. On the other hand, autophagy may also promote cell death in several situations. (1) In some cases, for instance when apoptosis is compromised, autophagy may directly act as an alternative cell death pathway, namely type II programmed cell death 35. (2) During autophagy, catalase may also be selectively degraded, causing ROS accumulation and necrosis cell death 36. (3) Autophagy can also directly induce apoptosis. 1‐(1‐Benzyl‐5‐(4‐chlorophenyl)‐1H‐1,2,3‐triazol‐4‐yl)‐2‐(4‐bromophenylamino)‐1‐(4‐chlorophenyl) ethanol is reported to induce autophagy‐dependent apoptosis in breast cancer cells both in vitro and in vivo 26. Human immunodeficiency virus type 1 (HIV‐1) envelope (Env) glycoproteins are proved to be able to induce autophagy‐dependent apoptosis in HIV‐1 infection 37. Similar results are also observed when citreoviridin (a mycotoxin and potent inhibitor of mitochondrial ATPase) is used in HepG2 cells, YM155 (a small molecule suppressor of Survivin) in prostate cancer cells and cyathin Q (a new cyathane type diterpene) in HCT116 cells 24, 25, 27. However, the mechanism of autophagy‐dependent apoptosis has not been well elucidated.

In the present study, we demonstrate that SM1044 induces autophagy‐dependent apoptosis in DLBCL cell lines. Additionally, we observed that SM1044 degrades the antiapoptosis protein Survivin through autophagy. Interestingly, YM155 (Survivin inhibitor) can also induce autophagy‐dependent apoptosis in prostate cancer cells 25. Survivin is a member of the IAPs family and is considered as one of the most potent apoptosis inhibitors. Of note, Survivin is expressed in a cell cycle‐dependent manner: it is minimally expressed in G1 phase, its expression increases in S phase, and reaches a peak in the G2/M phase. The knockdown of Survivin induces a G1 phase arrest in several different types of tumor cells 38, 39. Accordingly, our results show that a SM1044 treatment also induces a measurable increase in the proportion of DLBCL cells at the G1 phase of the cell cycle (Fig. S1A).

Survivin is shown to be acetylated by the histone acetyltransferase cAMP response element‐binding protein‐binding protein (CBP) or p300/CBP 40. Acetylation of lysine 129 in Survivin is responsible for its nuclear localization, while deacetylation of lysine 129 promotes its nuclear export. Since Survivin is reported to be less stable in the nucleus 41, increased acetylation of Survivin may suppress its nuclear export and promote its degradation. In agreement with this hypothesis, histone deacetylase (HDAC) inhibitor SAHA not only induces the acetylation of Survivin and its nuclear translocation, but also reduces the stability of Survivin in breast cancer cells 30. Here, we observed that the acetylation of Survivin is significantly increased after the addition of SM1044. Meanwhile, inhibiting p300/CBP rescued the degradation of Survivin. These results further support the hypothesis that the acetylation of Survivin accelerates its degradation.

Here we show that Survivin is degraded by SM1044 through autophagy, whereas the published data on the effect of SAHA suggested an acetylation‐dependent degradation of Survivin through the proteasome. It is therefore possible that the degradation of acetylated Survivin is dependent on site‐specific acetylation. Indeed, SAHA and SM1044 treatment could lead to different site‐specific acetylations of Survivin, which would result in different fates of the acetylated protein.

Next, we showed that acetylated Survivin interacts with LC3‐II and is degraded by autophagy, leading to the induction of apoptosis. In agreement with our findings on the role of Survivin in inhibiting autophagy and apoptosis, it was previously reported that the upregulation of Survivin by the C‐C motif chemokine ligand 2 (CCL2) inhibited autophagic cell death 42. In that study, the authors also found that Survivin could interact with LC3. Interestingly, they found that, although the expression of Survivin was upregulated by CCL2, the interaction between Survivin and LC3 was decreased by CCL2. These results are in line with our proposed mechanism which suggests that the interaction between Survivin and LC3 promotes Survivin's degradation through autophagy. However, the authors did not elucidate why and how Survivin interacted with LC3, nor did they explore the relationship between the Survivin‐LC3 interaction and the autophagic cell death inhibition.

In addition, we observed that ULK1 activation, one of the first steps of autophagy initiation, is induced by SM1044. Phosphorylation plays an important role in regulating the activity of ULK1. mTOR directly phosphorylates ULK1 at the inactivating site (Ser 757) to inhibit autophagy. AMPK directly phosphorylates ULK1 at the activating sites (Ser 317 or Ser 777) to induce autophagy 43. Moreover, AMPK can also inactivate mTOR, thus indirectly activating ULK1 44. In the present work, we observe that SM1044 activates ULK1 through the AMPK pathway instead of the mTOR pathway. AMPK is mainly regulated by two factors: the ratio of AMP/ATP and the intracellular level of Ca^2+^ 45. When the ratio of AMP/ATP rises, the activity of AMPK is enhanced by a LKB1‐dependent phosphorylation. Likewise, when the intracellular level of Ca^2+^ increases, CaMKK2 is activated, which subsequently leads to the activation of AMPK. In this study, we found that SM1044 activates the AMPK‐ULK1 axis which is mediated by CaMKK2.

It has been reported that THC induces the accumulation of ceramide, which leads to the AMPK‐dependent autophagy via activating CaMKK2 33. Interestingly, under this condition, autophagy also promotes apoptosis. Moreover, ART has been reported to induce ceramide in *Plasmodium falciparum* parasites, which plays a key role in the antimalarial activity of ART 34.

Ceramide has been shown to induce autophagy in several different ways: (1) inhibiting Akt, thus inducing autophagy through the suppression of the Akt‐mTOR signaling pathway 46; (2) activating JNK, which phosphorylates Bcl2 and releases Beclin1 from Bcl2 thereby inducing autophagy 47; and (3) suppressing the expression of nutrient transporter proteins, which results in AMPK‐mediated autophagy and cell death under nutrient‐rich conditions 48. In the present study, we found that the ART derivative SM1044 induces AMPK‐dependent autophagy through de novo synthesis of ceramide, since both the inhibitor of ceramide (S1P) and the inhibitor of de novo synthesis of ceramide (l‐cycloserine) reversed the effects of SM1044 (Fig. 5E–G). Ceramide synthase, which locates in the endoplasmic reticulum (ER), is responsible for catalyzing the de novo synthesis of ceramide. Interestingly, a fluorescent ART derivative mainly locates to the ER 49. Therefore, whether SM1044, the new derivative of ART, induced the de novo synthesis of ceramide directly or indirectly through ceramide synthase needs to be further investigated.

In conclusion, we synthesized a new water‐soluble derivative of ART, called SM1044, and demonstrated its ability to trigger autophagy and an autophagy‐dependent apoptosis. Furthermore, we unraveled the mechanism underlying the SM1044‐induced autophagy‐dependent apoptosis. Upon SM1044 treatment, we observed enhanced acetylation of Survivin, which in turn favors its interaction with LC3‐II and promotes the degradation of Survivin by autophagy. Additionally, we also provided evidence that SM1044 also activates AMPK, which is a key player in autophagy‐dependent apoptosis, by inducing de novo synthesis of ceramide.

These properties of SM1044 explain why SM1044 affects DLBCL cell survival in vitro and in vivo and argue in favor of its use in anti‐DLBCL therapeutic approaches.

## Conflict of Interest

None declared.

## Supporting information


**Figure S1.** Inhibitory effect of SM1044 on SU‐DHL‐4 cells. (A) SU‐DHL‐4 cells were treated with the indicated concentrations of SM1044 for 24 h. The proportions of cells at each phase of the cell cycle were determined by flow cytometry (mean ± SEM, *n* = 3). (B) SU‐DHL‐4 cells were treated with the indicated concentrations of SM1044 for 24 h. Mitochondrial membrane potential (MMP) was measured by flow cytometry (mean ± SEM, *n* = 3). **P < 0.01, ***P < 0.001.**Figure S2.** Autophagy is induced prior to apoptosis by SM1044. SU‐DHL‐4 cells were treated with the indicated concentrations of SM1044 for 6 or 24 h, respectively. Representative electron microscopy photomicrographs are shown. The green triangles indicate autophagosome, the yellow triangles indicate autolysosome, and the red arrows point to the apoptosis bodies.**Figure S3.** Autophagy inhibitors reverse the inhibitory effect of SM1044 in SU‐DHL‐4 cells. SU‐DHL‐4 cells were pretreated with CQ or Baf A1 for 1 h, followed by SM1044 treatment for another 24 h. Cell viability was measured by CCK‐8 (mean ± SEM, *n* = 3). ***P < 0.001.**Figure S4.** No significant changes were observed in the activity of LKB1 or ATP level after the addition of SM1044. SU‐DHL‐4 cells were treated with SM1044 for the indicated time courses. The expression of p‐LKB1 was detected by western blot (A) and the level of ATP was detected by an enhanced ATP assay kit (mean ± SEM, *n* = 3) (B). NS, no significance.
