# Supplementary material for: Induction of autophagy and autophagy‐dependent apoptosis in diffuse large B‐cell lymphoma by a new antimalarial artemisinin derivative, SM1044
Source: Cancer Med. 2017 Dec 26;7(2):380–96. doi: 10.1002/cam4.1276 (PMC5806110; doi:10.1002/cam4.1276)

Induction of autophagy and autophagy-dependent apoptosis in Diffuse large B-cell lymphoma by a new anti-malarial artemisinin derivative, SM1044

Additional Files

Supplementary Figure 1 Inhibitory effect of SM1044 on SU-DHL-4 cells. (**A**) SU-DHL-4 cells were treated with the indicated concentrations of SM1044 for 24 h. The proportions of cells at each phase of the cell cycle were determined by flow cytometry (mean±S.E.M, n=3). (**B**) SU-DHL-4 cells were treated with the indicated concentrations of SM1044 for 24 h. Mitochondrial membrane potential (MMP) was measured by flow cytometry (mean±S.E.M, n=3). ** $p<0.01$, *** $p<0.001$.

Supplementary Figure 2 Autophagy is induced prior to apoptosis by SM1044. SU-DHL-4 cells were treated with the indicated concentrations of SM1044 for 6 h or 24 h, respectively. Representative electron microscopy photomicrographs are shown. The green triangles indicate autophagosome, the yellow triangles indicate autolysosome and the red arrows point to the apoptosis bodies.

Supplementary Figure 3 Autophagy inhibitors reverse the inhibitory effect of SM1044 in SU-DHL-4 cells. SU-DHL-4 cells were pre-treated with CQ or Baf A1 for 1 h, followed by SM1044 treatment for another 24 h. Cell viability was measured by CCK-8 (mean±S.E.M, n=3). *** $p<0.001$.

Supplementary Figure 4 No significant changes were observed in the activity of LKB1 or ATP level after the addition of SM1044. SU-DHL-4 cells were treated with SM1044 for the indicated time courses. The expression of p-LKB1 was detected by

western blot (**A**) and the level of ATP was detected by an enhanced ATP assay kit (mean±S.E.M, n=3) (**B**). NS, no significance.

Supplementary Figure 1

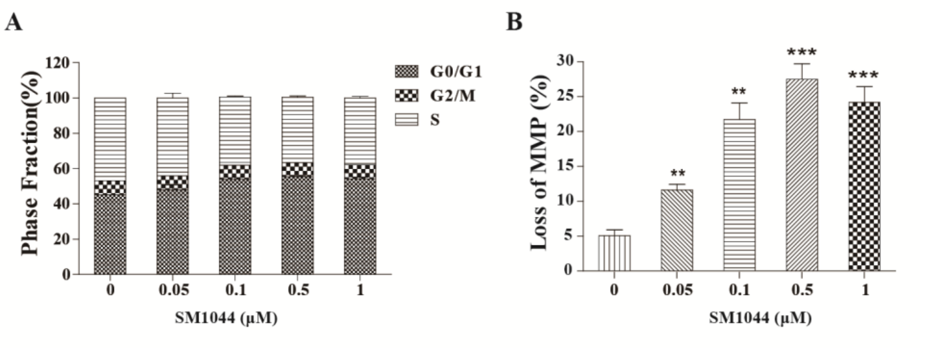

Supplementary Figure 2

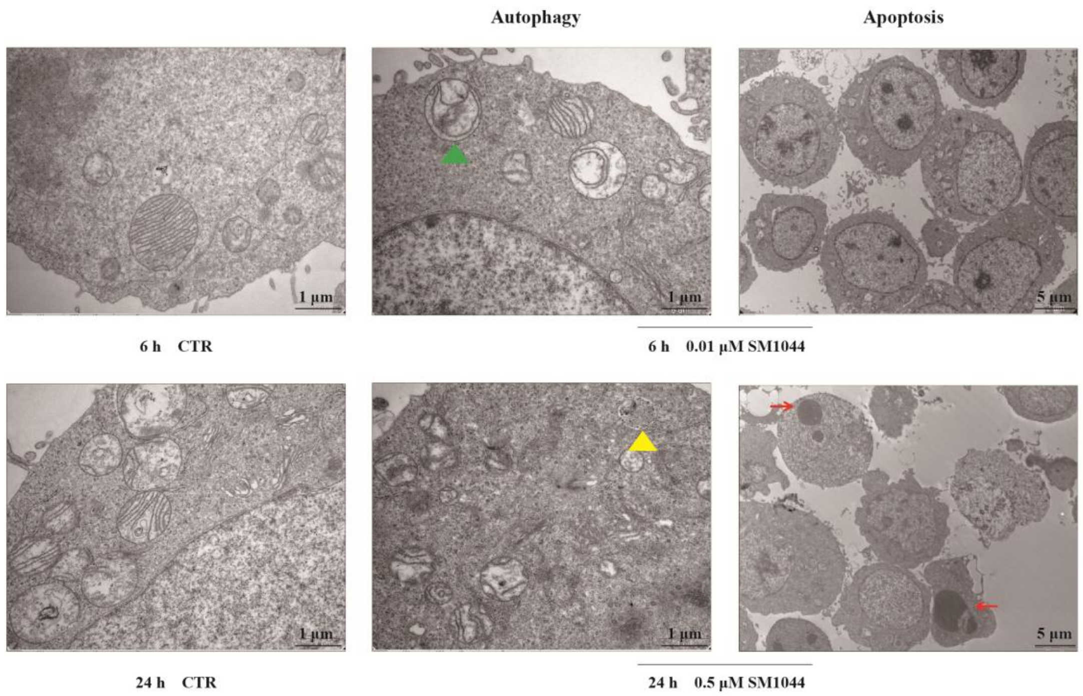

Supplementary Figure 3

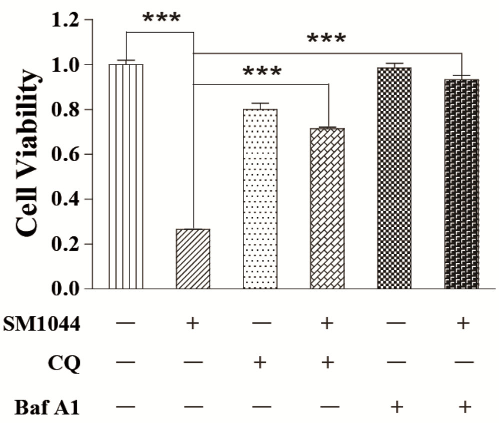

Supplementary Figure 4

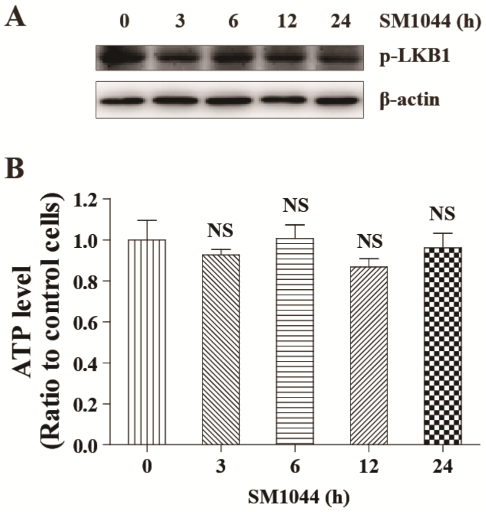

Supplement: Supplementary file 1 — Figure S1. Inhibitory effect of SM1044 on SU‐DHL‐4 cells. (A) SU‐DHL‐4 cells were treated with the indicated concentrations of SM1044 for 24 h. The proportions of cells at each phase of the cell cycle were determined by flow cytometry (mean ± SEM, n = 3). (B) SU‐DHL‐4 cells were treated with the indicated concentrations of SM1044 for 24 h. Mitochondrial membrane potential (MMP) was measured by flow cytometry (mean ± SEM, n = 3). **P < 0.01, ***P < 0.001.Figure S2. Autophagy is induced prior to apoptosis by SM1044. SU‐DHL‐4 cells were treated with the indicated concentrations of SM1044 for 6 or 24 h, respectively. Representative electron microscopy photomicrographs are shown. The green triangles indicate autophagosome, the yellow triangles indicate autolysosome, and the red arrows point to the apoptosis bodies.Figure S3. Autophagy inhibitors reverse the inhibitory effect of SM1044 in SU‐DHL‐4 cells. SU‐DHL‐4 cells were pretreated with CQ or Baf A1 for 1 h, followed by SM1044 treatment for another 24 h. Cell viability was measured by CCK‐8 (mean ± SEM, n = 3). ***P < 0.001.Figure S4. No significant changes were observed in the activity of LKB1 or ATP level after the addition of SM1044. SU‐DHL‐4 cells were treated with SM1044 for the indicated time courses. The expression of p‐LKB1 was detected by western blot (A) and the level of ATP was detected by an enhanced ATP assay kit (mean ± SEM, n = 3) (B). NS, no significance. [file CAM4-7-380-s001.pdf]
